# Barriers to timely transitions to comfort care in cancer patients: a review

**DOI:** 10.1097/MS9.0000000000003618

**Published:** 2025-07-22

**Authors:** Tenzin Tamdin, Sadikshya Bhandari, Samikshya Bhandari, Matthew Barbery, Ravi Bajwa

**Affiliations:** a Department of Internal Medicine, Resident, Danbury Hospital/Nuvance Health, Danbury, Connecticut, USA; b Department of Internal Medicine, Nepal Medical College, Kathmandu, Nepal; c Department of Internal Medicine, Danbury Hospital, Danbury/Nuvance Health, Connecticut, USA; d Department of Hematology/Oncology, Danbury Hospital, Danbury/Nuvance Health, Connecticut, USA

**Keywords:** comfort measures, end of life care, cancer patients, palliative care

## Abstract

**Introduction:**

“Comfort care” is a holistic approach for patients with terminal conditions, such as cancer, who are not expected to recover. It focuses on managing pain, among other end-of-life symptoms, and providing support to both the patient and their family during the dying process, which can last unpredictably from hours to days. End-of-life care concepts are often shaped by personal judgment and culture and thus lack a single consensus. This can lead to ambiguity in the term “comfort care” itself as well as create confusion in medical communication and treatment, making the transition to comfort care more challenging. In this review, we strive to evaluate the barriers preventing the timely conversion of end-stage cancer patients to comfort care. We also discuss the possible measures to limit these sensitive barriers to avoid futility in treatment and to ensure an ambiguity-free transition to ensure the best possible outcome for the patient. In this paper, we aim to systematically identify and categorize the key barriers that delay comfort care transitions in terminal cancer patients and to propose actionable strategies grounded in current evidence to address these challenges.

**Methodology:**

We conducted a literature search using PubMed and MEDLINE, covering studies published between January 2020 and March 2025. Search terms included a combination of MeSH terms and keywords such as “Palliative Care,” “Terminal Care,” “Hospice Care,” “Communication Barriers,” “Cultural Factors,” “Cancer,” and “Prognostic Uncertainty.” Eligible studies were peer-reviewed, published in English, and focused on adult cancer patients receiving palliative or end-of-life care, with a clear emphasis on barriers to timely transitions to comfort care. Studies unrelated to cancer, lacking a focus on barriers, or published in non-English languages were excluded. A total of 156 articles were identified; titles and abstracts were screened for relevance, followed by full-text review based on inclusion criteria. Data from the selected studies were analyzed using a thematic synthesis approach, in which two authors independently categorized findings under three main themes: prognostic uncertainty, communication challenges, and cultural factors. Discrepancies were resolved through discussion.

**Findings:**

Based on our extensive search, we classified the barriers for transition to comfort care into three main headings: Prognostic uncertainty, Challenges to Communication, and cultural factors. High levels of distress were observed in both patients and healthcare providers due to prognostic uncertainty, which complicates the prediction of illness trajectories, negatively affecting quality of life and delaying the transition to comfort care. Communication challenges, such as short consultations, language barriers, and difficult conversations about care goals, were also prevalent. The COVID-19 pandemic exacerbated these issues; there was lingering apprehension surrounding end-of-life discussions. Additionally, cultural factors play a significant role in shaping patients’ perceptions of cancer, pain, treatment, and death. Family involvement in decision-making varies across cultures, and systemic inequalities in access to palliative care disproportionately affect racialized groups.

**Conclusion:**

To overcome these barriers, the study emphasizes the need for effective policy-making to improve the quality of life for cancer patients during their care transition. It also becomes crucial to implement measures to improve communication protocols, validate culturally sensitive interventions, and routinely integrate palliative care in oncological practice early. We also need to work towards culturally appropriate interventions and strive to incorporate appropriate communication techniques to eliminate disparity for access to equitable palliative care.

## Introduction

Comfort care is a specialized branch of palliative care catered to providing care for patients with terminal conditions, including cancer, who are not expected to recover. It focuses only on symptom alleviation while improving quality of life during the last few days or weeks of life, without prioritizing cure, while providing physical, emotional, and psychological support for the patients and their families.

End-of-life care is a personal experience that is often shaped by the patient’s values and cultural background. Moreover, healthcare providers may approach comfort care in different ways depending on their training, philosophy, and personal judgments. This causes confusion in patients, making the transition to comfort care more complicated, causing delays in decision making and worsening patient outcome and quality of life. As such, it is paramount to have clear, compassionate discussions, as the emotional and logistic needs of both the patient and their families are addressed, based on a standardized universal framework for comfort care.

Despite being introduced since the early 1970s, there are still many barriers to the timely transition to comfort care in cancer patients. This literature review examines those barriers, while focusing on three distinct aspects: prognostic uncertainty, communication challenges, and cultural factors. Although discussed in three different headings, these issues are interconnected and significantly influence the decision-making process surrounding end-of-life care. This often leads to delays in implementing comfort-focused strategies that prioritize patient well-being and quality of life.

## Methodology

A literature review was conducted by searching PubMed and Medline databases, from 1 January 2018, to 31 December 2024. The search terms included “palliative care,” “end of life care,” “prognostic uncertainty,” and “cultural factors.” Inclusion criteria included peer-reviewed studies, published in English, consisting of cancer patients receiving palliative or end-of-life care, which directly addressed the barriers to timely transition to palliative or end-of-life care. This review has some limitations; these include language constraints, reliance on published literature, and subjective thematic interpretation.

## Discussion

We discuss the barriers to timely conversion to comfort care in end-stage cancer patients in detail in the following headings, as discussed briefly in Table 1.

**Table 1:** Barriers to comfort care

Barrier category	Challenges	Potential solutions
Prognostic uncertainty	Unknown disease trajectory, Emotional burden, The Lazarus effect.	The use of standardized prognostic training, the implementation of early goals-of-care (GOC) discussions, and the integration of early palliative care
Communication challenges	Time, expectations, language and cultural differences.	The SPIKES protocol, decision making, interpreters/cultural tailoring.
Cultural factors	Pain and end of life beliefs, family roles and functions, and healthcare mistrust.	Culturally appropriate health education, social workers and faith-based supporters, individualized communication.
System-level barriers	Lack of palliative care, ambiguous referral processes, practitioners’ reluctance.	Greater palliative integration, simple referral mechanisms, and telehealth availability.

### Prognostic uncertainty

Determination of prognosis is an inherently difficult and multifactorial decision in advance cancer care leading to uncertainty in decision making in both healthcare providers and patients while impacting end of life care and quality of life in patients^[1,2]^. Studies have shown that distress due to prognostic uncertainty worsens patients’ quality of life further during their end of life, with improvement seen with transition to hospice care^[1]^. Although understandable, studies have shown at least 77% of the patients are under moderate stress due to prognostic uncertainty which emphasizes the importance of the need for better communication strategies^[1]^. Further with changing guidelines, added comorbidities, as well as individualization of response to treatment, clinicians too face difficulties in prognostication for advanced cancer care^[3]^. This uncertainty worsens emotional burden and further complicates discussions regarding the end-of-life care causing delay in timely transition to comfort care^[3]^. The complexity of prognostication is further enhanced in certain cancer types, for instance, mesothelioma where caregivers experience high levels of distress due to ambiguity in treatment and individualized response^[4]^. Furthermore, the presence of clinical trials brings another level of complexity into the picture as patients have to decide between enrolling in trial interventions and receiving palliative care, which adds to the uncertainty of a final diagnosis and hope for improvement further adding to the stress^[5]^. The “Lazarus effect” – the unexpected survival – is itself a source of further uncertainty for both patients and oncologists^[3]^.

**HIGHLIGHTS**

- Comfort care
- End of life care
- Palliative care
- Cancer patients
- Barriers to comfort care

The issue of prognostic uncertainty is not limited to the interaction between the physicians and the patients. This uncertainty also affects the caregivers as well which needs to be addressed prior to discussion about alternative routes of management including hospice care^[6]^. The lack of clear and consistent information provision contributes to the overall distress and delays the conversion to comfort care. Therefore, prognostication and communication of the prognosis are essential for decision making and the formulation of the right care plan^[7]^. However, the low involvement of palliative specialists in the prognosis may pose a problem to this process^[7]^. Furthermore, the concept of “collusion” where the prognosis is kept from the patients, other specialities and caregivers leaves the decision-making process complicated^[7]^. This calls for more structured training for the healthcare professionals to enhance their interpersonal communication skills and resilience in conveying sensitive information^[7]^.

### Communication challenges

Effective communication is crucial in ensuring that patients are transferred to comfort care in a timely manner^[8,9]^. However, the studies have shown numerous communication issues that make it difficult to achieve this goal^[9,10]^. Short consultations between patients and healthcare workers in outpatient oncology departments can prevent the establishment of good rapport and trust that are necessary for these transitions. Good rapport between the patients and health care providers is crucial to the patients’ coping strategies as well as their levels of satisfaction with the provided care^[8]^. Miscommunication has further been linked with legal claims for medical malpractice as well^[9]^. Moreover, the process of shifting from prognostic conversation to comfort care conversation is quite challenging^[6]^. It is therefore important for the clinician to manage the family’s concerns and expectations from treatment and have honest conversations about the transition of care to hospice once treatment becomes futile^[6]^. Anderson et al stress the need to assure the patients and their families that end of life care will focus on making them as comfortable as possible, while explaining prognostication and transition of care.^[6]^. Shared decision making is critical in the process of informing patients about their care choices and influencing their shift to comfort care^[9]^. However, the time and process of goals-of-care (GoC) conversations is still ambiguous^[11]^.

The oncologists see GOC as a process that unfolds in three stages (initial, intermediate, and final) in accordance with the patient’s condition and emotional status. However, patients have different understandings on the timings and content of these discussions, which calls for individualized communication approaches. The change of focus from treatment to comfort is determined by the prognosis and the emotional readiness of the patient when shifting to hospice and comfort care.

In intensive care units (ICUs), conflicts related to decision making and communication issues within the multidisciplinary teams have been seen as major challenges. The involvement of family and patients in the decision-making process is a facilitator as well as a barrier, which makes shared decision-making in this context rather challenging. The organizational structure and resources also influence the effective incorporation of palliative care, which is a systemic barrier to ensuring the timely transition to comfort care^[10]^. In addition, language barriers are a significant determinant of prognosis and diagnosis discussions, which in turn delays the care transition, particularly for ethnic minority patients^[12]^.

To this end, palliative care providers, oncologists, including the multidisciplinary team involved in care must be aware of language barriers and try to include translators or providers who speak their native language while caring for patients with different linguistic backgrounds^[12]^. The COVID-19 pandemic has further worsened existing communication issues, which has prevented open discussions between healthcare providers and patients regarding care plans and end-of-life decisions^[13,14]^. Telehealth can provide some solutions, as it opens a new way of conversation and underscores the importance of relationship-based care^[14]^. The pandemic also led to increased psychological problems including loneliness and depression among cancer patients, which may lead to delayed transitions due to fear and isolation^[14]^.

### Cultural factors

Physician and patient cultural factors have an immense impact on how patients perceive cancer, pain, treatment, and death^[2,15,16]^. These factors can be real barriers to timely shift to comfort care^[15,16]^. For example, in Chinese immigrants, philosophical health beliefs that deem cancer pain as controllable and self-initiated with no need for medication are followed, together with cultural practices of pain reporting that are also low, which poses a problem to pain management and therefore worsen patients quality of life during end of life care^[15]^. Cultural beliefs and fears concerning the use of opioids result from cultural beliefs and fears regarding Western/modern medical practices^[15]^. In the same manner, there has been seen to be religious beliefs in African American women that may delay ovarian cancer diagnosis, affecting timely care transitions. Some of the beliefs identified include the belief that prayer can cure without the need for medical treatment, the fear that exposure to air or surgery can cause cancer to spread faster, the belief that cancer is a spirit or a curse from somewhere else, the belief that stress or worry makes the disease worse and the belief that health is destined. It means that these beliefs can hamper early detection and treatment, and therefore there is a need for culturally appropriate education and community based interventions to clear misconceptions and encourage early medical care^[16]^. Higher levels of religiosity and spirituality are linked with more advanced stages of diagnosis, which may affect treatment choices and the belief in healing without the use of medical intervention^[16]^. Health professionals in Colombia consider a patient’s medical condition and social environment when making care decisions; however, the fear of legal consequences has been seen to affect their practice, resulting in practices that are not consistent with the patient’s end-of-life wishes^[17]^. When caring for patients of indigenous groups, cultural factors play a significant role and it is important that physicians be sensitive to these cultural characteristics^[17]^.

Cultural factors also influence the role of family in decision making^[12,13]^. Family involvement and preference in comfort care plays an important role in shaping care provided to culturally diverse patients^[13]^. However, in practice, there have also been instances where the family has requested to keep the terminal diagnosis from the patient to avoid excess stress and fear during end of life care which has been shown to be a limiting factor in the transparent communication process^[18]^.

The COVID-19 pandemic has reiterated that cultural factors still play a big role in how patients are going to view and engage with comfort care during critical situations even more so in culturally diverse communities^[13]^. In India, the role of family decision making and cultural factors itself is more emphasized which can issue barriers in providing timely care to the patients^[9]^. The high cost of cancer treatment in India itself restricts the patient’s choice which may limit their chances of shifting to comfort care. Furthermore, the limited availability of palliative care in communities with poor access to care with infrastructure availability worsened by the pandemic, has made situations more complicated^[9]^. Algu et al in their paper discuss the systemic inequalities experienced by racialized groups in palliative care, including issues with communication and cultural safety that delay the transition to comfort care^[19]^. The study by Rosa et al further examines the need for culturally appropriate communication skills, especially in the light of the COVID-19 outbreak, which has introduced new challenges in the provision of palliative care^[14]^.

In addition, there are systemic barriers that hinder the progress of comfort care systems. These include limitations such as limited availability of palliative care services, especially in community settings, and a shortage of skilled personnel^[20,21]^. Lack of knowledge in palliative care among nurses and other healthcare professionals may pose a problem to the management of prognostic uncertainty and communication issues^[20]^. It also shows that the organizational structure of healthcare systems can be a problem as proven by the difficulty in incorporating palliative care in the intensive care setting and the role of the oncologists as the gate openers for the palliative care referral^[10,22]^. The misconception that palliative care is only for the end of life, the concern that patients will lose hope and the concern that the physician will break the therapeutic relationship also delays referral^[22]^. In addition, there are trust issues and power relationships between the healthcare providers that may hinder proper referral^[22]^. Patients and their families have to navigate through the healthcare system, which is a time consuming and tiring process and when transferred between teams, this makes the situation worse^[23,24]^.

## Possible Solutions

To overcome these systemic barriers, multi-faceted interventions should be adopted. These policies include; increasing palliative care training provided to healthcare providers, the establishment of formal communication plans to manage prognostic uncertainty and enhance the patient – provider interaction and the use of shared decision-making models that involve the patient and their families^[21,25,26]^. The involvement of the multidisciplinary team including oncology, palliative care and supportive care is crucial in managing the patient and making decisions. The use of a shared mental model among the care team can help to ensure that treatment plans are consistent with the patient’s goals and make the transition to hospice care easier^[27,28]^. The use of decision aids can enhance decision-making by identifying patient’s values and preferences through shared decision-making^[29]^. Human factors engineering can also be used in the design and implementation of decision aids to enhance patient centered cancer care^[27]^. Furthermore, the cultural and social factors should also be addressed through culturally appropriate interventions, population-specific communication strategies, and the empowerment of family caregivers^[15,16,19]^. The integration of palliative care into routine oncology practice will require the challenges and benefits to be addressed and the perceptions of healthcare providers and patients to be changed^[30,31]^. The use of standardized pathways and organizational models can help to reduce the barriers and enable the early integration of palliative care^[30]^. Telehealth can enhance the availability of care and enable communication, especially in rural areas or during outbreaks^[32]^. The need for more access to hospice care particularly at home cannot be overemphasized in order to ensure that patients get the best possible care at the end of life^[20,33]^.

## Conclusion

Prognostic uncertainty, communication difficulties, and cultural factors are important barriers to timely shift to comfort care for cancer patients. These barriers are interrelated and form a complex structure that requires a multifaceted approach to address. It thus becomes important to address these factors while considering the cultural and individual differences of the patients and their families through the improvement of communication, education, and the organization of the healthcare system. Future work should concentrate on the development and implementation of culturally appropriate interventions; improving communication, and enhancing the role of palliative care in regular oncological practice. Furthermore, studies should be conducted to explore the experiences of certain populations, for instance, adolescents and young adults with cancer, and the inequalities that exist within the system that affect vulnerable groups^[19,31,34]^. It is, therefore, crucial to develop and put in place effective policies to address these challenges so that the quality of life and health of cancer patients can be improved.

### Limitations

This review has several limitations. It included only English-language articles and used two databases, which may have led to the exclusion of relevant studies. The thematic synthesis involved some subjectivity, and we did not perform a formal quality assessment of included studies. Additionally, recent developments in palliative care may not be fully captured due to the search timeframe.

## Data Availability

Data sharing is not applicable to this article.

## Research registration unique identifying number (UIN)

UIN not available.
